# Production of Low‐Fat Mayonnaise With *Dunaliella salina* Protein: A Sustainable Alternative for Improved Sensory and Functional Properties

**DOI:** 10.1002/fsn3.71004

**Published:** 2025-10-28

**Authors:** Mohammad Hossein Hasanzadeh, Elham Mahdian, Esmaeil Ataye Salehi, Vahid Hakimzadeh

**Affiliations:** ^1^ Department of Food Science and Technology Qu.C Islamic Azad University Quchan Iran

**Keywords:** *Dunaliella salina*, low‐fat mayonnaise, ultrasound‐assisted extraction

## Abstract

The increasing demand for healthier food alternatives has led to the exploration of functional ingredients that can replace traditional fats in food products. *Dunaliella salina* , a microalga rich in proteins, presents a promising source for creating low‐fat formulations while maintaining desirable sensory and functional properties. This study aims to evaluate the potential of *Dunaliella salina* protein as a fat replacer in mayonnaise, focusing on its impact on physicochemical properties, rheological behavior, and sensory characteristics during a 10‐day storage period. The effect of the extraction method on physicochemical (pH, moisture content, emulsion stability, ash content, and FTIR spectroscopy) and rheological properties of *Dunaliella salina* protein was investigated. At the next step, the best method regarding to extracted protein properties was applied, and the protein was used in mayonnaise at 3 levels (1%, 3%, and 5%). Physicochemical, rheological, and sensory properties of mayonnaise were then evaluated. Results showed that the incorporation of *Dunaliella salina* protein significantly improved the viscosity and stability of the mayonnaise, with T3 (5% protein) exhibiting the highest enhancement in both functional and sensory properties. FTIR analysis revealed stronger protein‐lipid interactions in the protein‐enriched samples. Sensory tests demonstrated that T3 consistently outperformed other formulations in texture, color, odor, and overall acceptance. In conclusion, *Dunaliella salina* protein proves to be an effective fat replacer, enhancing both the sensory and functional properties of low‐fat mayonnaise, offering a sustainable and nutritionally enriched alternative for healthier food formulations.

## Graphical abstract

This study evaluated *Dunaliella salina* protein as a fat replacer in low‐fat mayonnaise. Ultrasound‐extracted protein showed superior emulsifying, antioxidant, and rheological properties. Mayonnaise with 5% protein exhibited the highest viscosity, stability, and sensory scores in texture, color, odor, and overall acceptance over 10 days. FTIR analysis confirmed enhanced protein–lipid interactions. Findings highlight *D. salina* protein as a sustainable ingredient to improve both the nutritional profile and the quality of reduced‐fat emulsions.

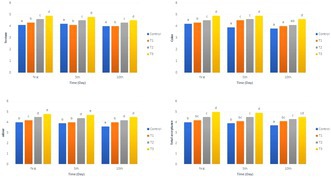

## 1 Introduction

In recent years, consumer preferences have experienced a notable shift toward healthier food alternatives, largely driven by heightened awareness of the adverse health effects associated with excessive fat consumption (Alsubhi et al. 2023). Per‐capita mayonnaise consumption remains high in many markets (1.9 kg/person·year in the U.S. and Canada; up to 5.1 kg/person·year in Russia) (EssFeed 2024). Mayonnaise, traditionally characterized by its high‐fat content, has seen a marked increase in demand for low‐fat or fat‐reduced variants. The formulation of low‐fat mayonnaise that maintains the same creamy texture and flavor as its full‐fat counterpart, while simultaneously addressing the growing consumer demand for healthier options, has become a key focus within the food industry. This trend underscores a broader societal shift toward achieving a balanced diet without compromising the sensory attributes of conventional food products (Gao et al. 2024). Consequently, the development of low‐fat mayonnaise incorporating alternative ingredients, such as functional proteins, presents a promising approach to fulfill health‐conscious consumers' nutritional and sensory needs (Srikanth et al. 2023).

Among alternative protein sources, the halophilic microalga *Dunaliella salina* has attracted attention for its rich nutritional profile and potential as a fat replacer in food formulations (Hyrslova et al. 2022). Found in hypersaline environments like the Dead Sea, this resilient alga thrives under extreme salinity, temperature, and light conditions, making it suitable for large‐scale cultivation (Ben‐Amotz 2019). *D. salina* contains 39%–61% protein by dry weight—up to 70% under optimal conditions—rich in essential amino acids such as leucine, lysine, and valine (Barbosa et al. 2023; Sousa et al. 2008). Beyond its high protein yield, *D. salina* protein is inherently amphiphilic—its balanced hydrophobic/hydrophilic residues give it excellent emulsifying capacity, comparable to or superior to many plant isolates. It also exhibits strong water‐holding and gelation properties, helping maintain mayonnaise's creaminess even when fat is drastically reduced. Moreover, *D. salina* carries natural antioxidants (notably β‐carotene) that can slow lipid oxidation in emulsions, and its GRAS status and ease of large‐scale cultivation make it a sustainable, safe functional ingredient for low‐fat formulations (Mosibo et al. 2024). Additionally, its natural antioxidants, notably β‐carotene, help reduce lipid oxidation (Pagels and Guedes 2023). With GRAS status granted by the FDA (Barros de Medeiros et al. 2022), *D. salina* represents a sustainable, safe, and functional ingredient for improving the texture, stability, and nutritional value of reduced‐fat food products (El‐Baz et al. 2017). Thus, *Dunaliella salina* offers a promising solution for developing healthier, low‐fat versions of traditional products like mayonnaise, by providing a sustainable and functional protein source that improves both the texture and nutritional composition of the final product.

The extraction of protein from microalgae involves several techniques, each influencing the quality and functionality of the final protein product. Common methods for protein extraction from *Dunaliella salina* include mechanical treatments such as ultrasound‐assisted extraction and high‐pressure homogenization, as well as chemical treatments involving water bath extraction and ethanol treatment. These methods are designed to break down the microalgal cell walls and release the protein in its most bioavailable form (Khalid et al. 2024; Nunes et al. 2024; Xia et al. 2019). The choice of extraction method significantly impacts the protein's solubility, emulsifying properties, and gelation ability, which are crucial for its application in food products (Chandran et al. 2024; Coelho and de las Mercedes Salas‐Mellado 2018). Understanding the effectiveness of these extraction methods is key to optimizing protein yield and functionality for specific food applications, such as in low‐fat mayonnaise.

The incorporation of *Dunaliella salina* protein into low‐fat mayonnaise formulations holds great promise due to the protein's functional properties, such as emulsification, gelation, and stability (Uribe‐Wandurraga et al. 2021). Emulsifiers are critical in the formulation of mayonnaise, where they help maintain a stable mixture of oil and water phases, preventing separation. Moreover, the addition of *Dunaliella salina* protein could potentially improve the rheological properties of low‐fat mayonnaise, such as its viscosity and texture, enhancing both its sensory qualities and nutritional (Taslikh et al. 2022). Therefore, investigating the effects of different extraction methods and protein concentrations on the final product's quality is crucial for optimizing its use in the food industry. Ouraji et al. (2020) investigated the use of enzymatically extracted faba bean protein as a partial substitute for egg yolk in reduced‐fat mayonnaise. Their findings showed that formulations containing equal amounts of faba bean protein and egg yolk powder (0.375%) or a higher proportion of faba bean protein (0.5%) with reduced egg yolk (0.25%) exhibited acceptable rheological, textural, and emulsion stability properties, indicating the potential of faba bean protein as a functional fat replacer (Ouraji et al. 2020). Heikal et al. (2023) explored the application of soy protein isolate (SPI) and nano‐formulated soy proteins, including nano soy protein isolate (NSPI) and nano glycinin (NGLY), as fat replacers in low‐fat mayonnaise. Their results demonstrated that the addition of 0.5%–1% NSPI and NGLY led to smaller and more compact oil droplets, enhanced emulsion stability, and improved sensory properties. Particularly, mayonnaise containing 0.5% NSPI and GLY received the highest sensory scores and maintained its quality over the storage period, suggesting the effectiveness of nano soy proteins in improving the texture and acceptability of low‐fat mayonnaise (Heikal et al. 2023). In a more recent study, Coelho et al. (2025) developed a plant‐based mayonnaise analogue using a combination of *Chlorella vulgaris* and *Saccharomyces cerevisiae* yeast protein extract. This mixed system demonstrated promising emulsion stability and enhanced the bioactive properties, including total phenolic content and antioxidant activity (Coelho et al. 2025).

The objective of this study is to explore the functional properties of *Dunaliella salina* proteins, particularly their rheological, emulsifying, and gel‐forming capabilities, and their potential application in low‐fat mayonnaise formulations. Additionally, the study aims to evaluate various protein extraction methods to determine the most efficient and effective process for optimizing these functional properties. Ultimately, this research seeks to contribute to the development of healthier, sustainable, and functional food products with enhanced nutritional value.

## 2 Material and Methods

### 2.1 Material

Microalgae ( *Dunaliella salina* ) was purchased from Persian Gulf Algae Development Technology Co. (Bushehr, Iran). Sunflower oil, salt, sugar, vinegar, and egg were purchased from local groceries in Mashhad, Iran. All reagents used for the analyses were analytical grade obtained from Sigma‐Aldrich Co. (St. Louis, MO, USA).

### 2.2 Protein Extraction

Protein extraction from *Dunaliella salina* was carried out using various methods, including mechanical and chemical treatments. The mechanical treatments involved ultrasonic‐assisted extraction, high‐pressure homogenization, and freeze–thaw cycles, while the chemical treatments utilized water bath extraction and ethanol (60%) solutions.

#### 2.2.1 Ultrasonic‐Assisted Extraction

The microalga ( *Dunaliella salina* ) was first hydrated by adding 1 g of dried algae to 10 mL of deionized water. The mixture was allowed to hydrate at room temperature (25°C) for 2 h, ensuring the algae cells were fully hydrated and then subjected to ultrasonic waves at a frequency of 40 kHz and power of 400 W. The ultrasound treatment was applied using a duty cycle of 50% (2 s on, 2 s off) for a duration of 10 min. After extraction, the sample was centrifuged at 11,200 rpm and 4°C for 15 min to separate the supernatant, which contained the protein extract (Kuhavichanan et al. 2018).

#### 2.2.2 Freeze–Thaw Cycles

*Dunaliella salina* powder was first hydrated at room temperature (25°C) for 2 h. The hydrated sample was then frozen at −20°C for 4 h and subsequently thawed at room temperature (25°C) for 2 h. Following thawing, the protein was extracted using the same centrifugation (11,200 rpm, 4°C, 15 min) and washing steps as described for the ultrasonic method (Pispas et al. 2024).

#### 2.2.3 Water Bath and Ethanol Extraction

The *Dunaliella salina* powder was mixed with a 60% ethanol solution in a 1:10 ratio, incubated in the water bath at 24°C for 2 h. The sample was then centrifuged and processed similarly to separate the protein and polysaccharides (Grossmann et al. 2018).

#### 2.2.4 Homogenization

*Dunaliella salina* powder was hydrated at room temperature (25°C) for 2 h. The hydrated suspension was then homogenized at 24,000 rpm for 20 min, maintaining the temperature at 25°C throughout the process. The homogenate was centrifuged at 11,200 rpm and 4°C for 15 min to recover the protein‐containing supernatant (Grossmann et al. 2018).

After extraction, the samples were dried using a freeze‐drying machine.

### 2.3 Physicochemical Analyses of Extracted Proteins and Mayonnaise

The pH of both the protein extract and mayonnaise was measured using a Hanna Instruments HI 221 pH meter, following (AOAC‐981 1981). Moisture content was determined gravimetrically by drying the samples at 105°C according to ISO 6496 (ISO‐6496 1983). The stability of the protein extract was evaluated by emulsion stability over one week, following ISO 3271 (ISO‐3271 1975), and ash content was measured by incinerating the sample at 550°C for 6 h, as per ISO 936 (ISO‐936 1998).

### 2.4 Protein Extraction Yield

The protein extraction yield was calculated by dividing the weight of the protein extract by the initial protein content of *Dunaliella salina* (dry weight basis). The total protein content was determined using the Kjeldahl method (AOAC‐978.04 2005), where the protein content was calculated by multiplying the nitrogen content by a factor of 6.25 (AOAC 2005).

### 2.5 Emulsifying Capacity and Emulsion Stability

The emulsifying capacity and emulsion stability of the protein extracted from *Dunaliella salina* were determined using the method described by Gupta et al. (2008). To measure the emulsifying capacity, 0.5 g of the protein extract and 50 mL of citrate buffer (0.5 M, pH adjusted to 5, 7, 9, and 10) were mixed with 10 mL of refined corn oil in a blender. The resulting suspension was mixed for 2 min and then transferred immediately to a 100 mL graduated cylinder (Gupta et al. 2008). To evaluate the emulsion stability, the total volume of the suspension and the height of the emulsion layer were measured over a 1‐week period (Chen et al. 2019). The emulsifying capacity was calculated using the following formula:

$$\begin{array}{cc} \text{Emulsifying capacity}\;\left(\%\right)=\; & (\text{Volume of the emulsion layer}/\\ & \text{Total volume of the mixture})\;\times \;100 \end{array}$$

### 2.6 Foaming Capacity and Foam Stability

The foaming capacity and foam stability of the protein extracted from *Dunaliella salina* were determined using the method described by Gupta et al. (2008). To measure the foaming capacity, 0.5 g of the protein extract and 50 mL of citrate buffer (0.5 M, pH adjusted to 5, 7, and 9) were added to the solution. The resulting suspension was mixed for 2 min and then quickly transferred to a 100 mL graduated cylinder. The volume of foam was recorded until half of the foam collapsed (Georgiou et al. 2024). The foaming capacity was calculated using the following formula:

$$\begin{array}{cc} \text{Foaming capacity}\;\left(\%\right)=\; & (\text{Volume of foam produced}/\\ & \text{Total volume of the solution})\;\times \;100 \end{array}$$

### 2.7 Mayonnaise Sauce Preparation

The mayonnaise samples were prepared according to the formulations listed in Table 1. T1 (20% oil) has equivalent fat content to the control (0% fat reduction), T2 (15% oil) corresponds to a 25% fat reduction, and T3 (10% oil) corresponds to a 50% fat reduction. The continuous phase consisted of a mixture of water, vinegar, sugar, salt, and the extracted protein, which was homogenized using a hand mixer for 2 min. The egg yolk, acting as the dispersed phase, was separated using an egg yolk splitter and then mixed with the oil. The protein mixture was then gradually incorporated into the egg yolk‐oil mixture. The entire mixture was blended for 8 min at room temperature to ensure a smooth and stable emulsion. The mayonnaise was then stored in sealed glass containers at 4°C for further analysis (Mirzanajafi‐Zanjani et al. 2019).

**TABLE 1:** Mayonnaise formulation.

Sample	Protein (%)	Oil (%)	Water (%)	Other ingredients^a^ (%)
Control	0	20	10	70
T1	1	20	9	70
T2	3	15	12	70
T3	5	10	15	70

^a^ Other Ingredients: “Q.S.” stands for Quantum Satis, meaning “sufficient quantity” to complete the formulation, including egg yolk, polysaccharide gums, vinegar, sugar, and salt.

### 2.8 Antioxidant Activity (DPPH Assay) for Mayonnaise

The antioxidant activity of the mayonnaise was assessed using the DPPH assay to evaluate its free radical scavenging potential. A 0.1 mL sample of mayonnaise with different protein concentrations (5, 25, 65, 125, 250 μg/mL) was mixed with 0.9 mL of DPPH solution (0.15 mM in methanol). The mixture was incubated in the dark at room temperature for 30 min. Absorbance was measured at 517 nm using a Shimadzu UV/Vis‐240 IPC spectrophotometer (Kyoto, Japan). The antioxidant activity was calculated based on the percentage of inhibition of DPPH radicals (Soltan et al. 2023).

### 2.9 Texture Analysis of Mayonnaise

The texture properties of the mayonnaise, including hardness, cohesiveness, and adhesiveness, were measured using a TA. XTplusC Texture Analyzer (Stable Micro Systems, Godalming, UK) equipped with a 10 kg load cell at room temperature. To assess the texture, 5°C mayonnaise was placed into a cylindrical container (26 mm inner diameter, 35 mm height) and smoothed at the top. A 12.7 mm diameter plastic cylinder (P/0.5–½″ Dia Cylinder Delrin, Stable Micro Systems) was used as the probe. The probe was inserted into the sample at a speed of 1 mm/s for a distance of 10 mm and then retracted at the same speed to its original position. Hardness was determined as the positive peak force during penetration, while adhesiveness was defined as the negative peak force during the withdrawal of the probe, indicating the resistance force. Cohesiveness was calculated as the ratio of the area under the curve during the second compression cycle to that of the first compression cycle, providing a measure of how well the sample holds together (Schädle et al. 2022).

### 2.10 Rheological Properties

The rheological properties of the protein extracts and mayonnaise were measured using a rheometer (MCR301, Anton Paar). The measurement was carried out using a plate‐plate geometry (PP25‐SN50999), with a constant strain applied to assess the flow behavior and viscosity of the samples. The samples were analyzed in the temperature range of 25°C, and the frequency sweep was conducted from 100 Hz to 0.01 Hz, with a logarithmic frequency scale. To model the non‐Newtonian behavior of the samples, the flow data were fitted using the power law and Herschel‐Bulkley models. The storage modulus (G′) and loss modulus (G″) were recorded to evaluate the elasticity and viscosity of the samples, respectively. Additionally, the damping factor (tan δ) was used to describe the energy dissipation in the samples, with complex viscosity (η) determined from the measured values. The deflection angle and torque were recorded for each sample, providing further insight into the rheological characteristics of the emulsions. Data were collected for each sample at multiple intervals, and the analysis was conducted to assess the viscoelastic properties and texture stability over time (Kumar et al. 2021).

### 2.11 FTIR

FTIR spectroscopy was performed to investigate potential interactions between the components of the mayonnaise. The samples were mixed with KBr powder at a mass ratio of 1:100. The mixture was ground into a fine powder and then pressed into transparent pellets. FTIR spectra were recorded using a Bruker Alpha FTIR spectrometer (Bruker Corporation, Billerica, MA, USA). The spectra were obtained by averaging 64 scans at a resolution of 4 cm^−1^, within the wavenumber range from 4000 to 400 cm^−1^. KBr powder was used as a baseline reference. The data were analyzed using the OMNIC software (Thermo Fisher, Waltham, USA) to identify the characteristic functional groups and possible interactions between the components of the mayonnaise (Yalmanci et al. 2023).

### 2.12 Sensory Analysis

The organoleptic properties of the mayonnaise, including aroma, color, texture, and overall acceptance, were evaluated by a trained sensory panel consisting of 15 panelists on Days 0, 5, and 10 of storage using the 5‐point hedonic scale. The panelists were trained in sensory evaluation techniques and were instructed to assess specific attributes of the mayonnaise. Aroma was rated for intensity and pleasantness, color for uniformity and brightness, texture for smoothness and consistency, and overall acceptance based on the combined sensory attributes. Samples were presented in random order to avoid bias, and evaluations were conducted in a controlled environment, ensuring that all external factors were consistent. The results were statistically analyzed to identify significant changes over the storage period (Mohammadi et al. 2024).

### 2.13 Statistical Analysis

Data were analyzed using two‐way analysis of variance (ANOVA) to assess the effects of time (0, 5, and 10 days) and treatment type on different attributes. The significance level was set at 0.05. When significant differences were found, post hoc pairwise comparisons were performed using Tukey's HSD test to determine which specific groups differed significantly. The analysis included two repetitions per sample, and the mean scores for each attribute were used for further statistical evaluation.

## 3 Result and Discussion

### 3.1 Extraction Efficiency

Among the evaluated methods (Table 2), ultrasound treatment demonstrated the highest protein extraction yield, with an average of 55.50% ± 0.70%, which was significantly higher than the other treatments (*p* < 0.05). This high efficiency can be attributed to the cavitation phenomenon generated by ultrasound waves, which effectively disrupts the cell wall, facilitates the release of intracellular contents, and enhances the interaction between proteins and the extraction medium. Homogenization, with a yield of 46.5% ± 2.10%, ranked second. While it also relies on mechanical force to disrupt cell structures, its intensity and uniformity of disruption appear to be lower than that of ultrasound. The freeze–thaw method yielded 36.50% ± 0.70%, suggesting a lower efficiency, possibly due to partial cell damage caused by ice crystal formation, which may not be sufficient to release all proteins (Berrouane et al. 2022). Finally, the aqueous bath with −60°C ethanol resulted in the lowest extraction yield (27% ± 1.40%). This method, primarily based on protein denaturation and precipitation, may have led to protein loss or reduced recovery efficiency. Overall, these findings highlight the significant impact of extraction method on the efficiency of protein recovery from *Dunaliella salina* , emphasizing the importance of selecting an appropriate technique for maximizing yield. In the study by Ferreira et al. (2023), the extraction yield using ultrasound‐assisted extraction for proteins and other bioactive compounds from *Dunaliella salina* was reported to be 62.8%, demonstrating the efficiency of the ultrasonic method (Ferreira et al. 2023). In the study by Berrouane et al. (2022), four different pretreatment techniques were compared for the extraction of C‐phycocyanin (C‐PC) from Spirulina platensis dry biomass, namely, freeze–thaw (F/T), enzymatic (EE), ultrasound (US), and pulsed electric field (PEF). The results showed that the ultrasound (US) pretreatment method provided the highest C‐PC yield of 129.50 ± 7.78 mg/g after 30 min of sonication at 40 kHz frequency. The freeze–thaw (F/T) pretreatment yielded a lower amount of C‐PC (Berrouane et al. 2022).

**TABLE 2:** Protein extraction efficiency.

Extraction method	Efficiency %
Ultrasound	55.5 ± 0.70^a^
Homogenization	46.5 ± 2.10^b^
Freeze–thaw	36.5 ± 0.70^c^
Aqueous bath with −60°C ethanol	27 ± 1.40^d^

*Note:* Values are expressed as mean ± standard deviation (*n* = 2). Different superscript letters within each column indicate statistically significant differences (*p* < 0.05) according to Tukey's HSD test.

### 3.2 Protein Characteristic

#### 3.2.1 Physicochemical Characteristic and Antioxidant Activity

According to Table 3 the extraction method significantly affected protein stability, pH, ash content, and antioxidant activity (*p* < 0.05), while moisture content showed no significant differences. The stability of protein extracts significantly varied depending on the extraction method (*p* < 0.05). Ultrasound‐extracted proteins (PDs1) exhibited the highest stability (95.5% ± 0.71%), followed by homogenization (PDs2: 93% ± 1.40%). The freeze–thaw method (PDs3: 89% ± 1.41%) and aqueous ethanol extraction (PDs4: 82% ± 1.40%) resulted in significantly lower stability values. The superior stability in PDs1 can be attributed to the effective disruption of cell walls and minimal protein denaturation by ultrasound, preserving the functional integrity of proteins. The pH values ranged from 4.55 to 5.25, with a significant increase observed in PDs4 (5.25 ± 0.04) compared to the other samples (*p* < 0.05). The relatively lower pH in PDs1 and PDs2 (4.55 ± 0.07 and 4.65 ± 0.05, respectively) may reflect fewer chemical modifications during extraction, while the higher pH in PDs4 could be due to alterations caused by ethanol and cold‐induced changes affecting the protein matrix or residual components. There were no statistically significant differences in the moisture content among the samples (*p* > 0.05), although a decreasing trend was observed from PDs1 (31.50% ± 0.70%) to PDs4 (28.75% ± 1.06%). This trend may be associated with the nature of the extraction process, where harsher or solvent‐based methods could lead to reduced water retention in the final protein product. Ash content significantly decreased across the samples, with PDs1 showing the highest mineral content (2.90% ± 0.20%) and PDs4 the lowest (1.70% ± 0.10%) (*p* < 0.05). The variation in ash levels may reflect differences in the efficiency of cell disruption and the degree to which inorganic compounds co‐extracted with the protein matrix. Ultrasound likely facilitated greater release of intracellular minerals compared to other methods. Antioxidant activity, as measured by DPPH radical scavenging, was highest in PDs1 (74.39% ± 0.87%) and lowest in PDs3 (55.79% ± 0.41%) (*p* < 0.05). The greater activity in PDs1 indicates better preservation or enhanced availability of antioxidant peptides, likely due to the non‐destructive nature of ultrasound extraction. Conversely, freeze–thaw and ethanol‐based methods may have led to degradation or loss of such functional components (Feng et al. 2022). Ultrasound extraction also helps increase protein bioavailability and digestibility by modifying protein structure, leading to the formation of bioactive peptides with potential health benefits, such as antioxidant and antihypertensive activities. Additionally, ultrasound extraction improves thermal stability, making the extracted proteins more suitable for food applications (Pandita et al. 2024). Similar to the findings of Xia et al. (2019), who demonstrated that *Dunaliella salina* protein can yield antioxidant peptides through ultrasound‐assisted extraction and gastrointestinal digestion, our study also highlights the potential of this protein for functional food applications. This suggests that *Dunaliella salina* , when processed using ultrasound, can provide not only functional proteins but also bioactive peptides, making it a promising ingredient for healthier, nutritionally enriched food products (Xia et al. 2019).

**TABLE 3:** Physicochemical and antioxidant properties of protein samples extracted from *Dunaliella salina* using different methods.

Protein obtained from different extraction methods	Stability (%)	pH	Moisture (%)	Ash (%)	DPPH scavenging activity (%)
PDs1*	95.50 ± 0.71^a^	4.55 ± 0.07^b^	31.50 ± 0.70^a^	2.90 ± 0.2^a^	74.39 ± 0.87^a^
PDs2	93 ± 1.40^b^	4.65 ± 0.05^b^	31.25 ± 0.30^a^	2.65 ± 0.07^b^	66.58 ± 0.82^b^
PDs3	89 ± 1.41^c^	4.90 ± 0.10^b^	29.50 ± 0.71^a^	2.40 ± 0.01^c^	55.79 ± 0.41^d^
PDs4	82 ± 1.40^d^	5.25 ± 0.04^a^	28.75 ± 1.06^a^	1.70 ± 0.10^d^	61.45 ± 2.05^c^

* PDs1: ultrasound, PDs2: homogenization, PDs3: freeze–thaw cycle, and PDs4: aqueous bath with −60°C ethanol. Values are expressed as mean ± standard deviation (*n* = 2). Different superscript letters within each column indicate statistically significant differences (*p* < 0.05) according to Tukey's HSD test.

#### 3.2.2 Functional Properties

According to Table 4, the emulsifying and foaming properties of protein samples extracted from *Dunaliella salina* were significantly influenced by the extraction method employed. These functional properties are critical in determining the suitability of protein ingredients for various food and pharmaceutical applications, particularly those involving emulsions and foams. The EC was observed in the ultrasound‐extracted protein (PDs1: 76% ± 1.41%), which was significantly greater than all other samples (*p* < 0.05). This superior emulsification ability can be attributed to the efficient cell disruption by ultrasound, leading to better solubilization and unfolding of proteins that expose hydrophobic groups, thus enhancing interfacial activity. Homogenization (PDs2) and freeze–thaw (PDs3) yielded moderate EC values (69.37% ± 0.88% and 61.75% ± 1.06%, respectively), while the aqueous ethanol method (PDs4) resulted in the lowest EC (49.50% ± 0.70%). The reduced emulsifying capacity in PDs4 may result from protein denaturation or aggregation during extraction, which limits its ability to interact with oil–water interfaces. Despite the differences in EC, ES remained statistically similar among PDs1 and PDs3 (*p* > 0.05), with values above 80%, indicating that once emulsions were formed, they maintained their structure relatively well in these samples. PDs4, however, showed a significantly lower ES (74% ± 1.41%), suggesting that the structural and functional integrity of the proteins extracted by ethanol treatment might have been compromised, affecting their capacity to stabilize emulsions over time. Ultrasound extraction also produced the highest foaming capacity (PDs1: 81.50% ± 2.12%), followed by homogenization (PDs2: 71% ± 1.41%), freeze–thaw (PDs3: 62% ± 2.80%), and ethanol treatment (PDs4: 41.50% ± 2.12%) (*p* < 0.05). In comparison with Mohammadi et al. (2024), who showed that amaranth protein isolate (API) emulsifying capacity (EC) varied from 62.20% at pH 4.0 to 80.5% at pH 2.0, and emulsion stability (ES) from 48.50% to 84.10%. Notably, ultrasound‐extracted DS protein (PDs1) achieved an EC of 76%, surpassing API's performance at neutral pH and approaching its optimum at pH 2.0. Likewise, ES for PDs1–PDs3 remained above 80%, mirroring the highest stability (84.1%) reported by Mohammadi et al., whereas PDs4 showed a lower ES (74% ± 1.40%), similar to the 48.5% minimum observed for API at pH 4.0. These results indicate that the choice of extraction method can modulate the interfacial activity of microalgal proteins, delivering emulsifying and stabilizing properties on par with those of plant‐derived APIs under their optimal conditions (Mohammadi et al. 2024). Foaming capacity is closely related to protein solubility, surface hydrophobicity, and molecular flexibility, all of which are enhanced in ultrasound‐treated proteins. The marked decline in FC for PDs4 further highlights the detrimental impact of harsh solvent‐based extraction on the protein's functional performance. In contrast to FC, foam stability exhibited less pronounced variation among PDs1 to PDs3, with no statistically significant differences observed (*p* > 0.05). These samples maintained foam structures effectively, with FS values around 74%–76%. However, PDs4 showed significantly lower foam stability (70.70% ± 1%), likely due to weaker film‐forming ability and reduced viscoelasticity of proteins after ethanol extraction. The findings clearly demonstrate that ultrasound extraction (PDs1) is the most effective method in preserving and enhancing the emulsifying and foaming functionality of proteins from *Dunaliella salina* . In contrast, the aqueous ethanol extraction (PDs4) significantly impairs these functional attributes. These differences are likely due to the varying degrees of protein structural integrity and surface activity retained under different extraction conditions. The choice of extraction method significantly influenced the functional properties of *Dunaliella salina* proteins. Ultrasound extraction (PDs1) resulted in the highest emulsifying and foaming capacities, which are critical for the formulation of stable emulsions like mayonnaise. The high emulsifying capacity of PDs1 may be attributed to better solubilization and protein unfolding, enhancing interfacial activity. On the other hand, the aqueous ethanol extraction (PDs4) produced proteins with lower emulsifying and foaming capacities, likely due to protein denaturation, which reduces their ability to interact with oil–water interfaces. Zhang et al. (2024) highlighted that ultrasound aids in protein solubility and structural rearrangement, improving antioxidant activity, which aligns with our results showing that ultrasound extraction enhances emulsifying and foaming properties. Both studies emphasize that ultrasound is effective in preserving protein integrity and functional performance, making it a promising method for producing proteins with improved functionality (Zhang et al. 2024). According to a review by Nunes et al. (2024), various extraction techniques, including ultrasound‐assisted extraction, significantly enhance the emulsifying capacity (EC) and foaming capacity (FC) of microalgal proteins. Ultrasound extraction, in particular, was found to exhibit superior performance in both EC and FC, attributed to efficient cell disruption, protein solubilization, and the exposure of hydrophobic groups, which improve interfacial activity. These findings align with our study, where ultrasound extraction of *Dunaliella salina* proteins resulted in the highest EC (76%) and FC (81.50%), confirming the positive impact of ultrasound on the functional properties of microalgal proteins, particularly in applications that require emulsification and foaming (Nunes et al. 2024). The superior stability of emulsions and foams in the PDs1 sample, compared to PDs4, highlights the importance of extraction methods in determining protein functionality. Ultrasound extraction preserved the proteins' structural integrity, leading to stable emulsions with high EC and ES values. In contrast, the ethanol‐extracted proteins (PDs4) exhibited lower functional performance, likely due to denaturation during extraction, which impaired the protein's ability to stabilize emulsions and foams.

**TABLE 4:** Emulsifying and foaming properties of protein samples extracted from *Dunaliella salina* using different extraction methods.

Protein obtained from different extraction methods	EC (%)	ES (%)	FC (%)	FS (%)
PDs1	76 ± 1.41^a^	82.66 ± 0.94^a^	81.50 ± 2.12^a^	76.38 ± 1.90^a^
PDs2	69.37 ± 0.88^b^	80.90 ± 1.28^a^	71 ± 1.41^b^	75.73 ± 1^a^
PDs3	61.75 ± 1.06^c^	80.50 ± 0.7^a^	62 ± 2.8^c^	74.50 ± 0.70^a^
PDs4	49.50 ± 0.70^d^	74 ± 1.41^b^	41.50 ± 2.12^d^	70.70 ± 1^b^

*Note:* PDs1: ultrasound, PDs2: homogenization, PDs3: freeze–thaw cycle and PDs4: aqueous bath with −60°C ethanol. Values are expressed as mean ± standard deviation (*n* = 2). Different superscript letters within the same column indicate statistically significant differences (*p* < 0.05) according to Tukey's HSD test.

#### 3.2.3 Rheological Properties

**FIGURE 1:**
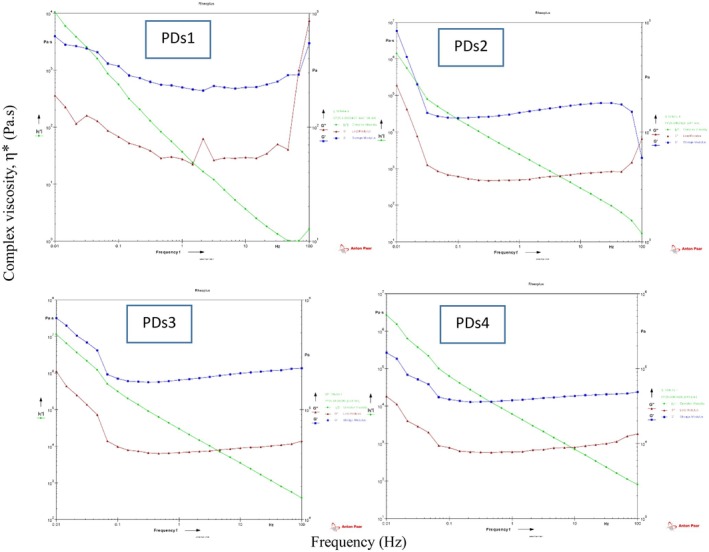
Viscoelastic properties of protein samples extracted from *Dunaliella salina* using different extraction methods; PDs1: ultrasound, PDs2: homogenization, PDs3: freeze–thaw cycle, and PDs4: aqueous bath with −60°C ethanol.

According to the rheological analysis of the protein extracts (Figure 1), significant differences were observed in the viscoelastic behavior of proteins obtained by different extraction methods. The key rheological parameters assessed included the storage modulus (G′), loss modulus (G″), and complex viscosity (|η|), providing insights into the elastic and viscous nature of the protein solutions. Protein extracted using ultrasound exhibited a typical viscoelastic profile with G′ consistently higher than G″ across the frequency range, indicating a dominant elastic behavior. The complex viscosity decreased with increasing frequency, displaying shear‐thinning behavior, which is desirable for many food applications (Guvendiren et al. 2012). The relatively moderate G′ and G″ values, along with a balanced tan δ (G″/G′), suggest a well‐structured, moderately strong gel‐like system with good deformation resistance and stability. The homogenized protein sample demonstrated the highest values for both G′ and G″ among all treatments, indicating a significantly stronger viscoelastic network. The dominance of G′ over G″ throughout the frequency range also confirmed a more solid‐like, elastic nature. This could be attributed to the mechanical shear during homogenization, which promotes extensive unfolding and interaction among protein molecules, resulting in a stronger gel matrix (Savadkoohi and Kasapis 2016). Complex viscosity values were also notably high, suggesting a dense internal structure. Proteins extracted via the freeze–thaw method displayed very high G′ and |η| values at low frequencies, but with a more gradual increase in G″, suggesting a highly elastic but possibly brittle structure. The strong G′ dominance indicates substantial protein–protein aggregation, likely caused by ice crystal formation and water removal during the freezing process (Bhatnagar et al. 2007). Although structurally rigid, this sample may exhibit lower flexibility under stress. This method yielded proteins with the lowest G′ and G″ values, indicating weak viscoelastic behavior and minimal network formation. The intersection of G′ and G″ occurred at lower frequencies, and the tan δ values were relatively high, signifying a predominantly viscous system. The reduced protein functionality in this case may stem from ethanol‐induced denaturation and reduced hydration, limiting the ability of proteins to form a cohesive network. The rheological behavior of the protein extracts clearly reflects the impact of extraction technique on protein structure and functional integrity (Moreira et al. 2025). Homogenization (PDs2) produced the most structured and elastic gel network, followed by freeze–thaw (PDs3) and ultrasound (PDs1). In contrast, aqueous ethanol extraction (PDs4) resulted in significantly weaker structural characteristics.

### 3.3 Mayonnaise Characterization

#### 3.3.1 Physicochemical and Antioxidant Activity Analysis

Based on Table 5, pH values of mayonnaise samples were significantly influenced by both storage time and formulation (*p* < 0.05). Initially, samples with higher protein and lower fat contents (especially T3) showed significantly higher pH values. For instance, T3 (5% protein, 10% fat) had the highest initial pH (5.05), while the control (0% protein, 20% fat) had the lowest (4.25). This is likely due to the buffering effect of proteins and reduced acid retention in low‐fat formulations. Over time, all samples experienced a pH decline, but the rate differed. Control and T1 showed the most pronounced decrease by day 10 (pH ~3.75), likely due to lower buffering capacity and higher susceptibility to lipid oxidation. In contrast, T2 and T3, with higher protein contents, maintained significantly higher pH levels, indicating better stability during storage. Increasing protein content in mayonnaise improved pH stability, while high fat and low protein formulations were more prone to acidification over time. In the study by Uribe‐Wandurraga et al. (2021), the pH values of microalgae‐fortified low‐fat emulsions showed slight variations during storage. The pH of emulsions containing *Chlorella* and *Dunaliella* increased slightly. The pH values ranged from 3.0 to 3.7 for low‐fat emulsions. These findings suggest that the incorporation of microalgae proteins helped stabilize the pH of the emulsions, particularly those fortified with *Chlorella* and *Dunaliella*, highlighting the role of protein fortification in enhancing pH stability over time (Uribe‐Wandurraga et al. 2021).

**TABLE 5:** Physicochemical and antioxidant properties of mayonnaise during storage.

Treatment	pH			Moisture (%)			Ash (%)			DPPH scavenging activity		
	0	5	10	0	5	10	0	5	10	0	5	10
Control	4.25 ± 0.07^j^	4.05 ± 0.07^l^	3.78 ± 0.11^k^	70.55 ± 0.64^j^	64.65 ± 0.21^k^	69.25 ± 0.35^l^	1.05 ± 0.07^j^	1.35 ± 0.07^l^	1.50 ± 0.0^k^	11.60 ± 0.14^j^	9.25 ± 0.35^l^	9.10 ± 0.14^k^
T1	4.45 ± 0.07^a^	4.38 ± 0.04^c^	3.75 ± 0.21^b^	69.15 ± 0.21^a^	67.25 ± 0.35^c^	65.35 ± 0.35^b^	1.25 ± 0.07^a^	1.48 ± 0.04^c^	1.85 ± 0.07^b^	20.25 ± 0.35^a^	19.70 ± 0.14^c^	19.10 ± 0.14^b^
T2	4.85 ± 0.07^d^	4.60 ± 0.14^f^	4.55 ± 0.07^e^	66.05 ± 0.07^d^	65.15 ± 0.21^f^	64.4 ± 0.57^e^	1.90 ± 0.14^d^	1.95 ± 0.07^f^	2.10 ± 0.14^e^	35.10 ± 0.28^d^	34.20 ± 0.28^f^	33.90 ± 0.14^e^
T3	5.05 ± 0.07^g^	4.75 ± 0.21^i^	4.85 ± 0.07^h^	65.70 ± 0.42^g^	64.15 ± 0.21^i^	63.2 ± 0.28^h^	2.05 ± 0.07^g^	2.40 ± 0.14^i^	2.45 ± 0.07^h^	49.70 ± 0.42^g^	46.2 ± 0.28^i^	43.75 ± 0.21^h^

*Note:* Different superscript letters within the same column indicate statistically significant differences (*p* < 0.05) according to Tukey’s HSD test.

Moisture content of mayonnaise significantly changed over the storage period (*p* < 0.05), and the extent of change varied with formulation. On Day 0, the control sample had the highest moisture content (70.55%), followed by T1 (69.15%), while T3 (with highest protein and water content) started with the lowest moisture (65.70%). This may be related to the initial formulation, where increased protein content led to stronger water binding within the emulsion, resulting in lower free moisture measurements. Over time, moisture content declined in all samples, mainly due to emulsion breakdown, water migration, and possible evaporation. The sharpest reduction was observed in the control, dropping to 64.65% on Day 5, but then increasing slightly on Day 10 (likely due to phase separation and sampling variability). In contrast, T3 showed the most consistent and gradual decline, ending at 63.20% on Day 10, which may reflect greater emulsion stability due to higher protein content. Overall, increasing protein in the formulation appeared to enhance water holding and structural stability, leading to a more controlled moisture loss over time.

Ash content of mayonnaise increased significantly (*p* < 0.05) over the 10‐day storage period, and the extent of this increase was directly related to the formulation, particularly protein content. On Day 0, ash levels ranged from 1.05% in the control to 2.05% in T3, which aligns with the increasing addition of protein‐rich ingredients in T1 to T3. Since proteins are natural sources of minerals, higher initial ash content in protein‐enriched samples (especially T2 and T3) is expected. Over time, ash content continued to rise in all samples. This can be attributed to moisture loss during storage, which concentrates the solid components, including minerals. The control showed the smallest increase (from 1.05% to 1.50%), while T3 reached the highest value (2.45%) by Day 10. The sharper increase in ash in protein‐rich samples may also suggest greater water loss and higher retention of protein‐bound minerals within the emulsion matrix. In conclusion, the results confirm that protein addition significantly increases ash content, and storage time further intensifies this effect due to moisture evaporation and solid concentration, particularly in high‐protein formulations.

DPPH radical scavenging activity was significantly affected by the formulation of the mayonnaise (*p* < 0.05), while no change was observed over storage time—all samples retained the same antioxidant activity throughout the 10‐day period. The control sample, which contained no added protein or antioxidant source, showed the lowest activity (11.60%), reflecting the minimal antioxidant capacity of the base formulation. As protein content increased across treatments, DPPH scavenging activity rose significantly. This trend indicates a strong positive correlation between protein enrichment and antioxidant activity, likely due to the presence of bioactive peptides or amino acids with radical‐scavenging potential in the protein source. The sharp increase observed in T3 suggests that higher protein levels not only enhance nutritional value but also provide functional antioxidant benefits. The results clearly demonstrate that protein fortification significantly improves the antioxidant capacity of mayonnaise, and these effects remain stable over time, supporting the formulation of functionally enriched mayonnaise products with improved oxidative stability. Similar to our results with *Dunaliella salina* protein, Mohammadi et al. (2024) reported improved emulsion stability, rheological characteristics, and sensory properties at higher amaranth protein isolate (API) concentrations in low‐fat mayonnaise. However, the primary advantages of using *D. salina* protein over API include its superior nutritional profile and inherent antioxidant activity due to β‐carotene content, as well as enhanced amphiphilic properties, resulting in potentially better emulsion stabilization. Nevertheless, *D. salina* protein may present challenges, such as higher extraction costs and possible impacts on product color and flavor, compared to the more neutral sensory attributes typically associated with API. Thus, further research optimizing extraction methods and sensory management strategies is recommended to enhance the practical application of *D. salina* protein in low‐fat mayonnaise formulations (Mohammadi et al. 2024).

#### 3.3.2 Texture Analysis

According to Table 6, texture attributes of mayonnaise—including hardness, cohesiveness, and adhesiveness—were significantly influenced (*p* < 0.05) by both the formulation and storage time. The differences observed highlight the functional role of protein enrichment in modifying and stabilizing the textural properties of the emulsions. At Day 0, hardness values increased proportionally with protein content, ranging from 12.25 N in the control to 18.65 N in T3 (5% protein). This trend continued over storage, with all samples showing gradual increases due to moisture loss and emulsion tightening. By Day 10, T3 reached the highest firmness (20.05 N), suggesting that higher protein levels strengthen the emulsion matrix, possibly through protein–protein and protein–lipid interactions that enhance structural rigidity. Cohesiveness also showed a significant formulation‐dependent pattern. Initially, T3 and T2 exhibited the highest cohesiveness (0.96 and 0.95), indicating a more elastic and structurally unified matrix, likely due to stronger internal bonding provided by higher protein concentrations. Over time, cohesiveness slightly decreased in all samples, more noticeably in low‐protein formulations (e.g., control and T1), possibly due to microstructural weakening or phase separation during storage. However, T3 maintained the highest cohesiveness even after 10 days (0.89), confirming greater internal structural integrity. Adhesiveness (negative force needed to detach the probe from the sample) was most pronounced in protein‐rich samples. At Day 0, the control had the lowest adhesiveness (−0.7 N.s), while T3 showed the highest (−1.45 N.s). This indicates that protein addition enhances interaction with surfaces, increasing stickiness, likely due to improved water retention and gel‐like consistency. Over storage, a slight decrease in adhesiveness was observed across all samples, possibly due to oil separation and water loss, yet T3 consistently retained the highest values, suggesting better structural and interfacial stability. Overall, protein enrichment in mayonnaise formulations significantly improved texture by increasing firmness, cohesiveness, and adhesiveness. These improvements were maintained over the 10‐day storage period, especially in T2 and T3, highlighting the dual role of proteins as structural and stabilizing agents in reduced‐fat or functional emulsions. The results support the application of protein fortification to enhance the sensory and physical quality of mayonnaise during shelf‐life. The results of Uribe‐Wandurraga et al. (2021) align with our findings in terms of the positive impact of protein fortification on the textural properties of low‐fat emulsions, particularly regarding firmness, cohesiveness, and adhesiveness. Both studies show that higher protein content improves the stability and structural integrity of emulsions over time. However, Uribe‐Wandurraga et al. observed that emulsions with Chlorella maintained more stable texture throughout storage, while in our study, all formulations, especially T3, experienced gradual increases in firmness due to moisture loss and emulsion tightening. This suggests that in our study, the protein‐enriched formulations may have enhanced network formation and structural stabilization more effectively. Additionally, Uribe‐Wandurraga et al. found Chlorella‐fortified emulsions to have more stable textural properties compared to other microalgae, while our results indicate higher protein content across all formulations leads to more consistent textural improvements (Uribe‐Wandurraga et al. 2021).

**TABLE 6:** Texture analysis of mayonnaise during storage.

Treatment	Hardness (N)			Cohesiveness			Adhesiveness (N.S)		
	0	5	10	0	5	10	0	5	10
Control	12.25 ± 0.35^j^	13.30 ± 0.42^l^	13.90 ± 0.14^k^	0.82 ± 0.04^j^	0.82 ± 0.02^l^	0.82 ± 0.04^k^	−0.70 ± 0.14^j^	−0.68 ± 0.03^l^	−0.59 ± 0.01^k^
T1	14.60 ± 0.14^a^	15.15 ± 0.21^c^	15.90 ± 0.14^b^	0.86 ± 0.02^a^	0.86 ± 0.01^c^	0.82 ± 0.02^b^	−0.95 ± 0.07^a^	−0.88 ± 0.02^c^	−0.78 ± 0.03^b^
T2	16.90 ± 0.14^d^	17.15 ± 0.21^f^	18.30 ± 0.42^e^	0.95 ± 0.07^d^	0.89 ± 0.01^f^	0.86 ± 0.02^e^	−1.25 ± 0.07^d^	−1.05 ± 0.07^f^	−1.15 ± 0.21^e^
T3	18.65 ± 0.21^g^	19.25 ± 0.35^i^	20.05 ± 0.35^h^	0.96 ± 0.06^g^	0.95 ± 0.07^i^	0.89 ± 0.01^h^	−1.45 ± 0.07^g^	−1.30 ± 0.14^i^	−1.33 ± 0.04^h^

*Note:* Different superscript letters within the same column indicate statistically significant differences (*p* < 0.05) according to Tukey’s HSD test.

#### 3.3.3 Rheological Behavior

**FIGURE 2:**
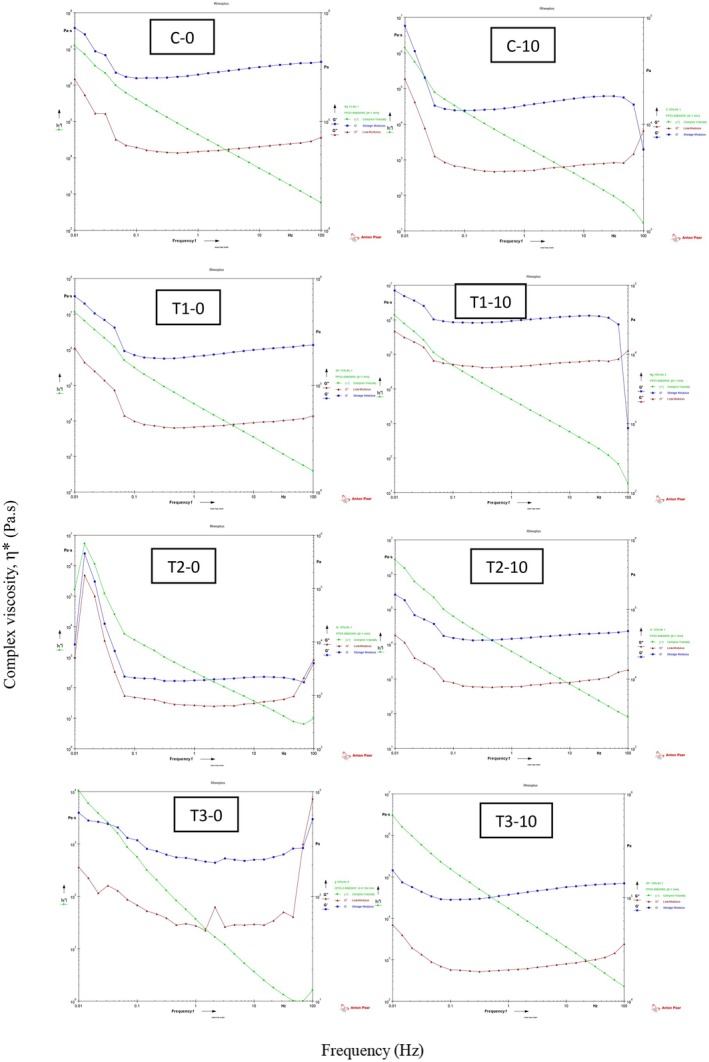
Viscoelastic properties of mayonnaise during storage; C‐0: Without added protein at Day 0, C‐10: Without added protein at Day 10, T1‐0: 1% protein at Day 0, T1‐10: 1% protein at Day 10, T2‐0: 3% protein at Day 0, T2‐10: 3% protein at Day 10, T3‐0: 5% protein at Day 0, and T3‐10: 5% protein at Day 10.

The rheological behavior of the control mayonnaise showed (Figure 2) a marked decline from Day 0 to Day 10 of storage. On Day 0, the sample exhibited a strong viscoelastic structure, with high storage modulus (G′ ≈ 3.5 × 10^5^ Pa) and complex viscosity (η ≈ 1.2 × 10^7^ Pa·s), indicating a stable, gel‐like emulsion. However, by Day 10, both G′ and η* decreased substantially (to ~5.8 × 10^3^ Pa and ~1.4 × 10^6^ Pa·s, respectively), accompanied by an increase in damping factor (tan δ > 0.3), reflecting a shift toward more viscous and less structured behavior. These changes suggest weakening of the internal emulsion network, likely due to moisture loss, pH drop, and possible phase separation over time. The results confirm that, without stabilizing agents such as protein, the texture and structural integrity of mayonnaise deteriorate significantly during storage. The rheological properties of T1 mayonnaise exhibited a notable enhancement over the 10‐day storage period. On Day 0, the sample displayed moderate viscoelasticity, with storage modulus (G′) values ranging from ~8.5 × 10^2^ Pa at high frequencies to ~8.3 × 10^4^ Pa at the lowest frequency, and a relatively low complex viscosity (~1.3 × 10^6^ Pa·s). In contrast, by Day 10, the rheological profile significantly improved, with G′ values increasing up to ~6.8 × 10^5^ Pa and complex viscosity reaching ~1.15 × 10^7^ Pa·s at low frequencies. The consistently low damping factor (tan δ < 0.25) throughout the test suggests a dominant elastic behavior on both days; however, the strengthening of G′ and η by Day 10 indicates a denser and more structured network formation. These improvements are likely due to protein–protein and protein–lipid interactions promoted during storage, enhancing the gel structure and stability of the emulsion. Overall, the results demonstrate that the T1 formulation maintained and even improved its structural integrity over time, in contrast to the control, and reflects the beneficial effect of moderate protein enrichment on the rheological performance of mayonnaise. The rheological analysis of sample T2 revealed a notable enhancement in its viscoelastic properties during the 10‐day storage period. On Day 0, T2 exhibited relatively weak gel‐like characteristics with low storage modulus (G′ ~2000 Pa) and high damping factor (tan δ > 0.3–0.5 in most frequencies), indicating a predominantly viscous nature. However, by Day 10, the structure had significantly strengthened, as reflected by a substantial increase in G′ values across all frequencies, reaching ~1.63 × 10^5^ Pa at the lowest frequency, along with a more stable and lower tan δ (mostly < 0.23), indicating a stronger elastic response. Additionally, the complex viscosity increased noticeably, confirming the formation of a more cohesive viscoelastic network. These rheological improvements are likely due to progressive interactions among proteins, fat, and aqueous phases over time, as well as structural stabilization enhanced by the moderate protein content (3%) and reduced fat level (15%) in the T2 formulation. Overall, these findings suggest that T2 develops a stable emulsion matrix with improved textural integrity over time. The rheological properties of the T3 mayonnaise underwent a remarkable enhancement from Day 0 to Day 10 of storage. On Day 0, the sample exhibited relatively weak gel‐like behavior, characterized by low storage modulus (G′) values starting from ~546 Pa at high frequencies and reaching only ~627 Pa at the lowest frequency. The damping factor (tan δ) values fluctuated around 0.2 to 0.3 at low frequencies, indicating a dominance of viscous over elastic behavior in some regions. However, by Day 10, a substantial structural improvement was observed, with G′ values increasing by nearly two orders of magnitude (up to ~1.83 × 10^5^ Pa at low frequencies), accompanied by increased complex viscosity (η) and a damping factor consistently below 0.26 in most frequency ranges. These changes reflect the development of a significantly stronger viscoelastic network, likely attributed to protein–protein and protein–water interactions over time, driven by the higher protein and moisture content in the T3 formulation. The pronounced increase in both moduli and decrease in tan δ over time suggest that T3 achieved the most stable and elastic emulsion structure among the samples, making it highly promising in terms of textural and rheological quality during storage. The enhanced emulsifying and foaming properties of PDs1 protein resulted in improved viscosity and emulsion stability in the mayonnaise formulations. Specifically, the protein‐enriched mayonnaise formulations demonstrated better structural integrity over time, reflected in higher G′ and η values in the rheological analysis. The findings of Uribe‐Wandurraga et al. (2021) are generally consistent with our study, though there are some differences in the storage duration and rate of improvement in rheological properties. Both studies show that microalgae proteins (such as *Arthrospira platensis* and *Dunaliella salina* ) significantly enhance the rheological behavior and stability of low‐fat emulsions, resulting in more stable emulsions with improved viscoelastic properties. However, Uribe‐Wandurraga et al. observed stabilization over a longer storage period (60 days), while our study found that protein‐enriched mayonnaise demonstrated noticeable improvements in rheological properties within a shorter storage period (10 days). This difference could be attributed to several factors. One major factor might be the type of emulsion system used. In their study, the focus was on low‐fat oil‐in‐water emulsions, which typically exhibit slower stabilization due to their more complex formulation and the properties of the fat phase. On the other hand, our study focused on mayonnaise formulations, which may have different viscoelastic properties and could respond more quickly to protein fortification, leading to faster improvements in rheology. Additionally, the extraction method of the microalgae proteins might play a role in the differing rates of stabilization. While Uribe‐Wandurraga et al. (2021) used biomass directly from microalgae, which could potentially contain a mixture of different proteins and other components, our study utilized proteins, which might have resulted in more efficient incorporation into the formulation and faster stabilization. Therefore, the results of both studies align in terms of the positive impact of microalgae protein fortification, but the storage time and rate of rheological improvement differ slightly between the two (Uribe‐Wandurraga et al. 2021). The findings of Metri‐Ojeda et al. (2022) regarding the rheological behavior of low‐fat mayonnaises are largely consistent with our study, but there are notable differences in the protein source and additives used, which may explain some of the observed variations. Both studies observed shear‐thinning (pseudoplastic) behavior in low‐fat mayonnaise formulations, where viscosity and consistency index (K) decreased as oil content was reduced. This is consistent with our findings, where a decrease in oil content similarly led to weaker rheological properties. Moreover, both studies indicate that protein enrichment plays a significant role in improving the rheological stability of low‐fat mayonnaises. Metri‐Ojeda et al. (2022) found that vegetable proteins, including soy protein isolate (SPI) and spirulina protein concentrate (SPC), helped mitigate viscosity loss, while our study showed that microalgae proteins, specifically those from Arthrospira platensis, also enhanced the viscoelastic properties of the formulations. However, the difference between the two studies lies in the use of additional thickeners. Metri‐Ojeda et al. employed sodium alginate to further enhance the viscosity of their formulations, whereas our study focused exclusively on microalgae proteins without the need for additional thickeners. This led to a quicker stabilization of rheological properties in our study. The microalgae proteins not only contributed to the improvement of viscoelastic properties but also resulted in faster stabilization in the mayonnaise formulation, compared to the slower stabilization observed by Metri‐Ojeda et al. (2022). Moreover, the rate of rheological improvement in our study was faster, possibly due to the different characteristics of microalgae proteins versus vegetable proteins, and the absence of additional stabilizers. In contrast, Metri‐Ojeda et al. (2022) reported that their emulsions required a longer time to stabilize due to the complex interactions between protein, sodium alginate, and reduced oil content. In conclusion, while both studies share similarities in the positive impact of protein fortification on the rheological properties of low‐fat mayonnaises, our study observed a faster stabilization effect with microalgae proteins and without the need for additional thickeners, which is a key difference between the two studies (Metri‐Ojeda et al. 2022). In comparison to Mohammadi et al. (2022), who showed that incorporating rice bran protein (RBP) into dairy desserts increased apparent viscosity (η_a_), storage (G′) and loss (G″) moduli, and reduced syneresis, our ultrasound‐extracted *D. salina* protein (PDs1) produced analogous rheological improvements in low‐fat mayonnaise. Specifically, PDs1 exhibited the highest zero‐shear viscosity (η₀) and longest relaxation time (λ) under the Cross model, indicative of a stronger protein network (Mohammadi et al. 2022). A recent study by Zakeri et al. (2025) demonstrated that using acetylated distarch adipate in ketchup significantly improved rheological and sensory properties, including viscosity, texture firmness, and structural stability. Similarly, in our study, incorporating *Dunaliella salina* protein led to remarkable enhancements in emulsion stability, viscosity, and sensory attributes such as texture and overall product acceptance. The primary advantage of *D. salina* protein compared to modified starch is its superior nutritional value, antioxidant properties, and improved emulsion stability resulting from its inherent amphiphilic characteristics. However, there are notable limitations, including the higher cost associated with protein extraction and its potential effects on the color and flavor of the final product compared to modified starches. Therefore, further research is recommended to optimize extraction methods for *D. salina* protein and to effectively manage its sensory impacts (Zakeri et al. 2025).

#### 3.3.4 FTIR

**FIGURE 3:**
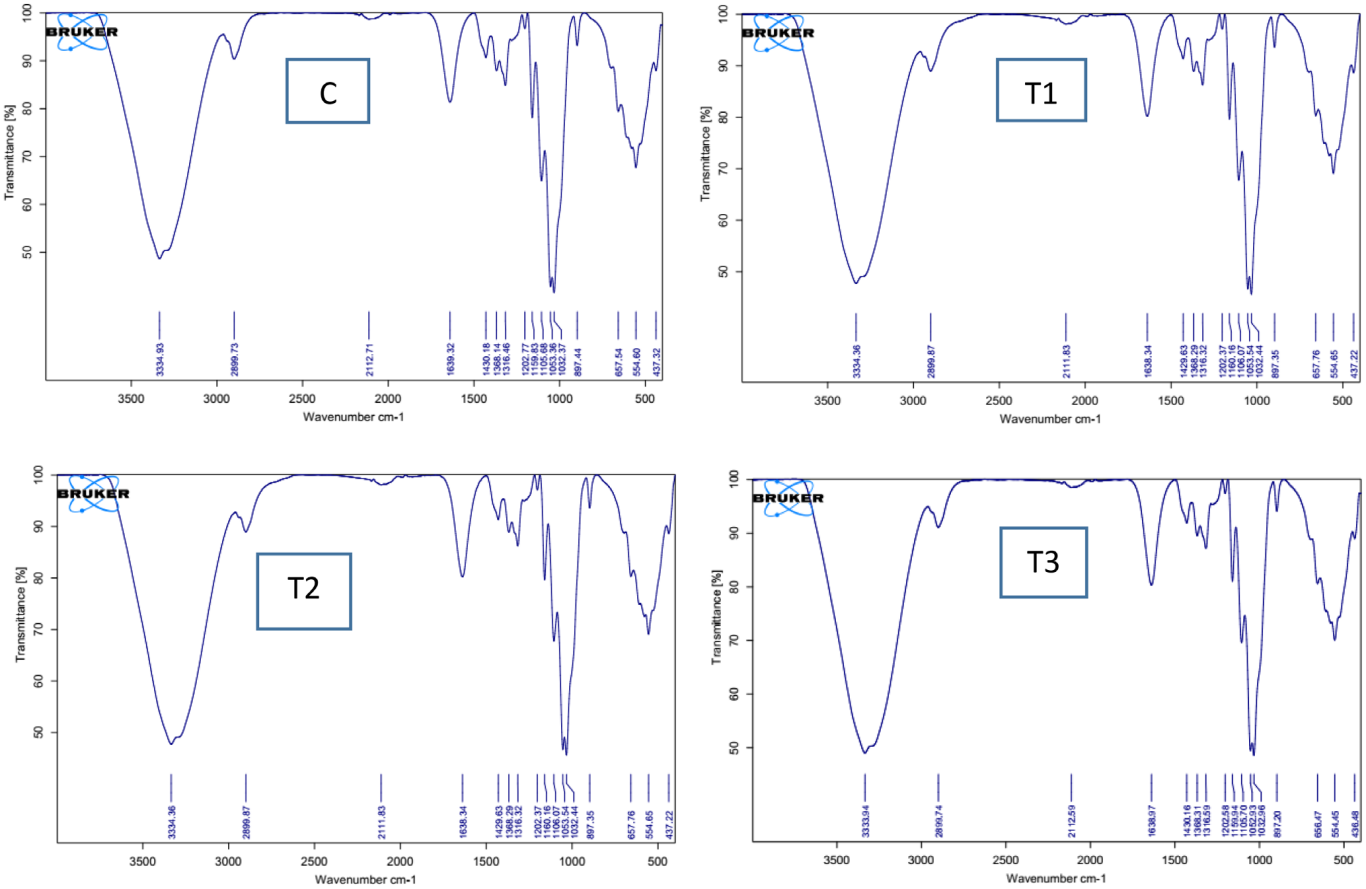
FTIR analysis of mayonnaise: C: Without added protein, T1: 1% protein, T2: 3% protein, T3: 5% protein.

The FTIR spectra of the four sauce samples (Figure 3) show distinct variations in the functional groups present, reflecting the differences in their chemical compositions. The Control sample exhibits prominent peaks around 3300 cm^−1^, which is characteristic of hydroxyl groups (–OH) and amide groups, typically found in lipids and proteins (Weidner et al. 2009). This peak suggests the presence of water and protein components, with a notable contribution from lipid structures. The peak around 1600 cm^−1^ corresponds to the C=C stretching vibration of unsaturated bonds, indicating the presence of unsaturated fatty acids in the lipid phase (Dev and Mukadam 2025). As the protein content increases in samples T1, T2, and T3, new peaks emerge, indicating significant changes in the chemical structure. For the T1 sample, subtle shifts in the spectra are observed, with new bands appearing around 1650 cm^−1^, which are attributed to C=O stretching vibrations, indicative of the amide group of proteins (Martinez‐Cuazitl et al. 2021). This suggests that the introduction of a small amount of protein leads to a noticeable but mild change in the sample's chemical structure, particularly influencing its protein content. In the T2 sample, the peaks at 1650 cm^−1^ and 1600 cm^−1^ become more intense, indicating a stronger presence of amide groups and C=C stretching, likely due to a higher concentration of protein (Ahmad and Ayub 2022). The increased intensity of these peaks suggests that the higher protein content in T2 induces more substantial interactions between the protein and other components, such as lipids, altering the molecular structure further compared to T1. For the T3 sample, the spectra exhibit even more pronounced shifts, particularly in the 1700 cm^−1^ region, associated with C=O stretching and amide groups, indicating a significant increase in the protein concentration (Rakipov et al. 2022). The intensity of the peaks in the 1600 and 1500 cm^−1^ regions also suggests the formation of a more complex protein‐lipid network, which is expected to contribute to a more stable and structured emulsion system. The increased intensity of the amide and C=O stretching peaks in T3 is a clear indication of stronger protein‐lipid interactions, which may have significant implications for emulsion stability and the overall texture of the final product. The FTIR spectra suggest that as protein content increases (from Control to T1, T2, and T3), there is a noticeable intensification in peaks related to amide groups (1650 cm^−1^), C=O stretching (1700 cm^−1^), and C=C bonds (1600 cm^−1^) (Wang et al. 2021). These changes indicate that protein addition enhances the formation of protein‐lipid interactions, which can impact emulsion stability. In particular, the formation of a stronger protein‐lipid network could improve the mechanical stability and texture of the sauces, as well as their sensory characteristics such as mouthfeel and viscosity. These findings align with the observed differences in texture, color, odor, and overall acceptance among the samples. The enhanced protein‐lipid interactions in T3 could explain its superior sensory characteristics, such as improved mouthfeel and texture, compared to lower protein content samples. This supports the hypothesis that the protein‐lipid network plays a crucial role in emulsion stability and sensory quality.

#### 3.3.5 Sensory Analysis

**FIGURE 4:**
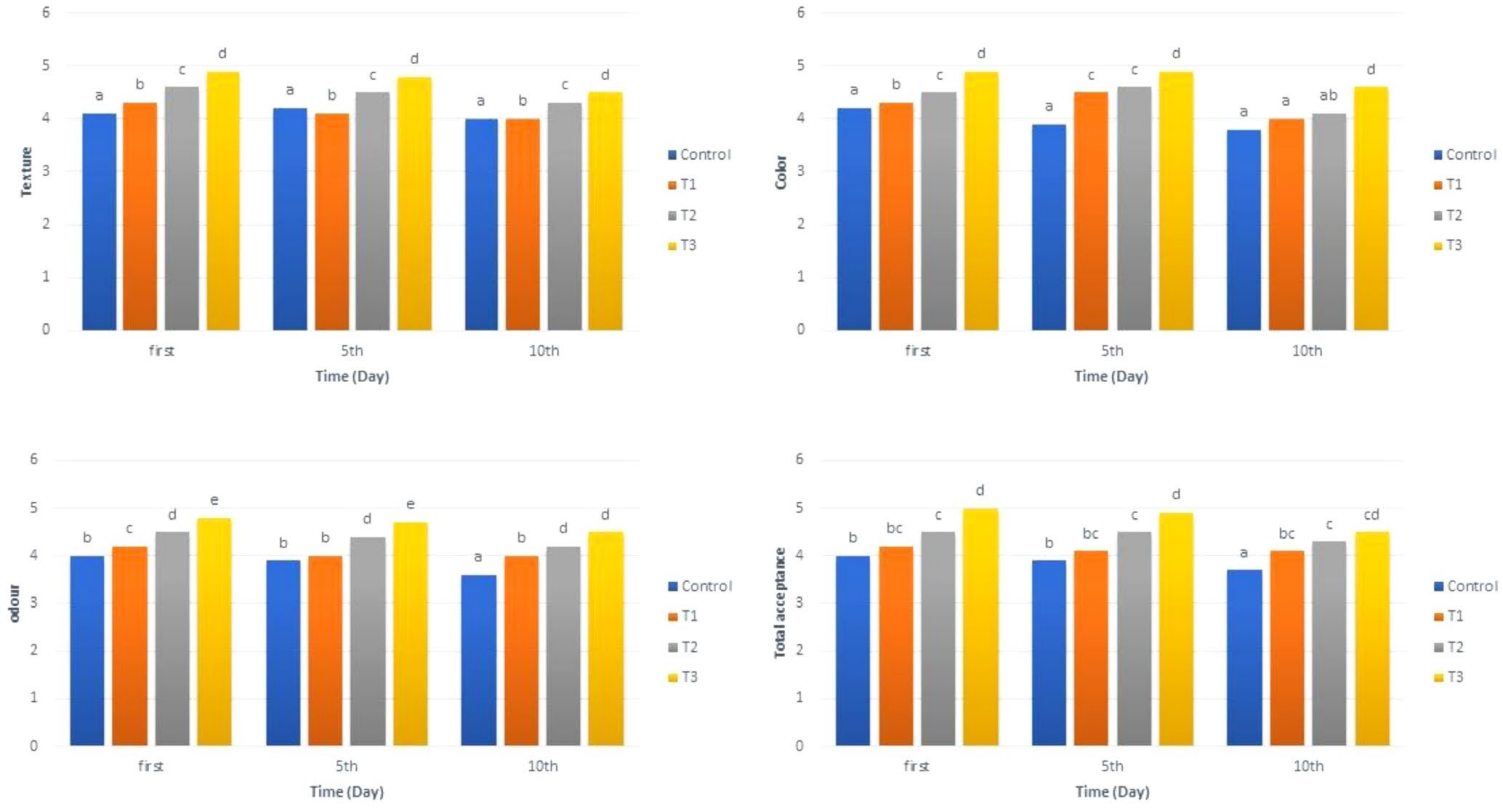
Sensory analysis of mayonnaise during storage; C: Without added protein, T1: 1% protein, T2: 3% protein, T3: 5% protein.

The sensory analysis results in Figure 4 show significant variations in the sensory attributes of the mayonnaise formulations over time. These changes are closely correlated with the physicochemical properties, such as texture, color, odor, and overall acceptance, highlighting the influence of protein content, oil concentration, and storage duration on the final sensory characteristics.

The texture of the sauces varied significantly across the formulations. Sample T3, which contained the highest protein content, exhibited the softest texture compared to the other formulations, especially on Day 0. This aligns with the moisture content and pH values, which were better retained in T3 due to the higher protein content, leading to a more stable emulsion structure. As storage time increased, the textural differences remained significant, with T3 consistently maintaining the best texture profile. This could be attributed to the interactions between proteins and water, which affect gel network formation and overall texture stability. The increased protein content in T3 helped maintain a more stable emulsion, reducing breakdown of texture over time, which was more pronounced in T1 and T2, where texture declined due to lower protein content and weaker emulsion stability. Significant differences in color were observed among the sauce formulations, particularly for T3, which exhibited the most vibrant color on Day 0. Over time, color differences became less pronounced, but the formulations with higher protein content, such as T2 and T3, maintained more appealing color characteristics. The pH stability observed in these formulations, particularly T3, may have played a key role in maintaining the color stability, as lower pH typically leads to more pronounced Maillard reactions and color degradation. As higher protein content in T2 and T3 contributed to better pH retention, these formulations showed more stable color properties compared to T1, which experienced greater color shifts due to the lower protein and pH values over time. Odor analysis indicated that T3, with the highest protein content, had the strongest odor on Day 0, and this odor intensity remained high throughout the storage period. This finding is consistent with the observed increased protein content and higher pH in T3, which likely enhanced the formation of volatile aromatic compounds. T1 and T2 also displayed significant changes in odor over time, with T2 showing more stability in odor compared to T1. The changes in odor can be explained by the interaction of protein content with storage time, which influenced the volatile compounds in the sauces. Higher protein levels in T2 and T3 likely led to the formation of more aromatic compounds during storage, improving the overall aroma profile of the sauces. Overall acceptance scores were significantly higher for T3, which consistently outperformed the other formulations across all days. This suggests that the combination of higher protein content, altered oil and water content, and better physicochemical stability in T3 resulted in a more favorable sensory profile, including better texture, color, and odor. T1 and T2 showed varying levels of acceptance, but they were generally lower than T3, supporting the notion that a balanced formulation, particularly with higher protein content, is crucial for improving the overall sensory quality of the sauces. Throughout the evaluation, the sensory changes in texture, color, odor, and overall acceptance closely correlated with the physicochemical properties of the sauces. For instance, the higher protein content in T3 contributed to better moisture retention and pH stability, which directly influenced the texture and color stability. Similarly, the higher protein content in T2 and T3 helped to stabilize odor and improved the overall acceptance of the sauces, as compared to T1, which had lower protein content and more rapid deterioration in texture and odor over time. The findings of Metri‐Ojeda et al. (2022) align with our study in terms of protein enrichment improving texture and overall acceptance of low‐fat mayonnaise. Both studies found that higher protein content enhanced the sensory properties, with T3 (the highest protein content) showing the best results in our study, similar to their findings. However, differences were observed in appearance and color stability: while Metri‐Ojeda et al. noted that spirulina protein concentrate caused a green color issue, our study showed that T3 maintained a more vibrant color. This difference in color stability may be due to variations in the protein extraction method or the presence of other ingredients. Metri‐Ojeda et al. used spirulina protein concentrate directly, while our study utilized proteins, which might have improved the color stability and minimized color change during storage. Additionally, both studies agree on the positive effect of protein content on texture, although Metri‐Ojeda et al. also highlighted oil concentration as a contributing factor to texture improvement. This suggests that the interaction between protein and oil concentration may be more prominent in their study, where the oil content ranged between 15% and 30%, while in our study, the oil content was lower and the emphasis was more on the protein source. Overall, while the general trends are consistent, differences in appearance and color stability indicate that the type of protein source used may influence these properties (Metri‐Ojeda et al. 2022).

Although our fortification of low‐fat mayonnaise with *D. salina* protein yielded encouraging improvements in texture, stability, and nutritional profile, some inherent constraints of this preliminary study should be noted. Specifically, the work was conducted at laboratory scale with a limited number of experimental replicates, short‐term storage conditions, and a narrow range of formulation levels. Building on these initial findings, we recommend the following avenues for future research:

1. Scale‐up and Process Optimization.
2. Expanded Sensory and Shelf‐Life Studies.
3. In Vivo and Nutritional Assessments.

## 4 Long‐Term Performance and Safety

These strategic directions will help validate and extend the applicability of microalgal protein fortification in functional food matrices, paving the way toward industrial implementation and health‐oriented product development.

## 5 Conclusion

This study highlights the successful incorporation of *Dunaliella salina* protein into low‐fat mayonnaise formulations, offering a promising alternative for developing healthier food products while maintaining desirable sensory qualities. The findings demonstrate that increasing protein concentrations significantly enhance both the functional and sensory properties of mayonnaise, improving texture, emulsifying capacity, and overall stability. The FTIR analysis confirmed that the incorporation of *Dunaliella salina* protein strengthened the protein‐lipid interactions, which played a crucial role in enhancing the structural integrity and texture of the product. The study emphasizes the importance of the extraction method in optimizing the physicochemical and rheological properties of the protein. While the selected extraction method proved effective in preserving the functional properties of the protein, further research into different extraction methods could provide additional insights into their impact on protein efficiency and the final product's performance. This gap in understanding the direct connection between extraction methods and mayonnaise performance will be an essential area for future investigations. Sensory evaluation revealed that T3 consistently outperformed the other formulations in terms of texture, color, odor, and overall acceptance, indicating that higher protein levels contribute significantly to sensory enhancement. These results suggest that *Dunaliella salina* protein is not only a sustainable and functional protein source but also a viable option for improving the nutritional profile of food products aimed at health‐conscious consumers. Overall, the incorporation of *Dunaliella salina* protein into mayonnaise represents a significant step toward the development of reduced‐fat, nutritionally enriched products with superior sensory qualities. This study lays the groundwork for future research exploring the application of microalgae‐derived proteins in various food formulations, contributing to the growing demand for healthier, functional, and sustainable food alternatives. Future studies should focus on refining extraction techniques and exploring their effects on both the sensory and physicochemical properties of food products, paving the way for more innovative and sustainable food solutions.

## Author Contributions

**Mohammad Hossein Hasanzadeh:** investigation (equal), writing – original draft (equal). **Elham Mahdian:** investigation (equal), resources (equal), supervision (equal), writing – review and editing (equal). **Esmaeil Ataye Salehi:** investigation (equal), resources (equal), supervision (equal), writing – review and editing (equal). **Vahid Hakimzadeh:** writing – review and editing (equal).

## Conflicts of Interest

The authors declare no conflicts of interest.

## Data Availability

The authors confirm that the data supporting the findings of this study is available within the article.

## References

[fsn371004-bib-0001] Ahmad, A. , and H. Ayub . 2022. “Fourier Transform Infrared Spectroscopy (FTIR) Technique for Food Analysis and Authentication.” In Nondestructive Quality Assessment Techniques for Fresh Fruits and Vegetables, 103–142. Springer.

[fsn371004-bib-0002] Alsubhi, M. , M. Blake , T. Nguyen , I. Majmudar , M. Moodie , and J. Ananthapavan . 2023. “Consumer Willingness to Pay for Healthier Food Products: A Systematic Review.” Obesity Reviews 24, no. 1: e13525. 36342169 10.1111/obr.13525 PMC10909406

[fsn371004-bib-0003] AOAC . 2005. Official Methods of Analysis. Association of Official Analytical Chemists.

[fsn371004-bib-0004] AOAC‐978.04 . 2005. “Official Methods of Analysis of AOAC International.” Method Number: 978.04. In: Rockville, MD, USA: AOAC International.

[fsn371004-bib-0005] AOAC‐981 . 1981. “(AOAC) 981.12: pH of Foods.” In: AOAC International (Association of Official Analytical Chemists).

[fsn371004-bib-0006] Barbosa, M. , L. G. Inácio , C. Afonso , and P. Maranhão . 2023. “The Microalga Dunaliella and Its Applications: A Review.” Applied Phycology 4, no. 1: 99–120.

[fsn371004-bib-0007] Barros de Medeiros, V. P. , W. K. A. da Costa , R. T. da Silva , T. C. Pimentel , and M. Magnani . 2022. “Microalgae as Source of Functional Ingredients in New‐Generation Foods: Challenges, Technological Effects, Biological Activity, and Regulatory Issues.” Critical Reviews in Food Science and Nutrition 62, no. 18: 4929–4950. 33544001 10.1080/10408398.2021.1879729

[fsn371004-bib-0008] Ben‐Amotz, A. 2019. The Alga Dunaliella. CRC Press.

[fsn371004-bib-0009] Berrouane, N. E. H. , F.‐S. Attal , A. Benchabane , et al. 2022. “Freeze‐Thaw‐, Enzyme‐, Ultrasound‐and Pulsed Electric Field‐Assisted Extractions of C‐Phycocyanin From Spirulina Platensis Dry Biomass.” Journal of Food Measurement and Characterization 16, no. 2: 1625–1635.

[fsn371004-bib-0010] Bhatnagar, B. S. , R. H. Bogner , and M. J. Pikal . 2007. “Protein Stability During Freezing: Separation of Stresses and Mechanisms of Protein Stabilization.” Pharmaceutical Development and Technology 12, no. 5: 505–523. 17963151 10.1080/10837450701481157

[fsn371004-bib-0011] Chandran, A. S. , P. Kashyap , and M. Thakur . 2024. “Effect of Extraction Methods on Functional Properties of Plant Proteins: A Review.” eFood 5, no. 3: e151.

[fsn371004-bib-0012] Chen, Y. , J. Chen , C. Chang , et al. 2019. “Physicochemical and Functional Properties of Proteins Extracted From Three Microalgal Species.” Food Hydrocolloids 96: 510–517.

[fsn371004-bib-0013] Coelho, M. S. , and M. de las Mercedes Salas‐Mellado . 2018. “How Extraction Method Affects the Physicochemical and Functional Properties of Chia Proteins.” LWT 96: 26–33.

[fsn371004-bib-0014] Coelho, P. , C. Serrano , N. Komora , and A. Raymundo . 2025. “From a Coriander Mayonnaise to a Vegan Analogue: Assessing pH and Salt Influence in a *Saccharomyces Cerevisiae* Yeast Protein Extract and *Chlorella Vulgaris* Mixed System.” Food 14, no. 4: 587. 10.3390/foods14040587 PMC11854552 40002031

[fsn371004-bib-0015] Dev, M. , and M. Mukadam . 2025. “Functional Group Profiling of Medicinal Plants Using FTIR Spectroscopy.”

[fsn371004-bib-0016] El‐Baz, F. K. , S. M. Abdo , and A. M. Hussein . 2017. “Microalgae *Dunaliella salina* for Use as Food Supplement to Improve Pasta Quality.” International Journal of Pharmaceutical Sciences Review and Research 46, no. 2: 45–51.

[fsn371004-bib-0017] EssFeed . 2024. “Top 10 Countries Consuming the Most Mayonnaise Per Capita.” https://essfeed.com/top‐10‐countries‐consuming‐the‐most‐mayonnaise‐per‐capita‐top‐10‐countries‐consuming‐the‐most‐mayonnaise‐per‐capita/.

[fsn371004-bib-0018] Feng, Y. , D. Yuan , B. Kong , et al. 2022. “Structural Changes and Exposed Amino Acids of Ethanol‐Modified Whey Proteins Isolates Promote Its Antioxidant Potential.” Current Research in Food Science 5: 1386–1394. 36110385 10.1016/j.crfs.2022.08.012 PMC9468495

[fsn371004-bib-0019] Ferreira, J. P. , M. Grácio , I. Sousa , A. Pagarete , M. C. Nunes , and A. Raymundo . 2023. “Tuning the Bioactive Properties of *Dunaliella salina* Water Extracts by Ultrasound‐Assisted Extraction.” Marine Drugs 21, no. 9: 472. 37755085 10.3390/md21090472 PMC10532918

[fsn371004-bib-0020] Gao, Y. , Y. Zhao , Y. Yao , et al. 2024. “Recent Trends in Design of Healthier Fat Replacers: Type, Replacement Mechanism, Sensory Evaluation Method and Consumer Acceptance.” Food Chemistry 447: 138982. 38489876 10.1016/j.foodchem.2024.138982

[fsn371004-bib-0021] Georgiou, D. , A. Charisis , A. Theocharidou , et al. 2024. “Foaming Properties of *Chlorella sorokiniana* Microalgal Biomass.” Colloids and Interfaces 8, no. 6: 66.

[fsn371004-bib-0022] Grossmann, L. , S. Ebert , J. Hinrichs , and J. Weiss . 2018. “Production of Protein‐Rich Extracts From Disrupted Microalgae Cells: Impact of Solvent Treatment and Lyophilization.” Algal Research 36: 67–76.

[fsn371004-bib-0023] Gupta, S. , G. Chandi , and D. S. Sogi . 2008. “Effect of Extraction Temperature on Functional Properties of Rice Bran Protein Concentrates.” International Journal of Food Engineering 4, no. 2: 107.

[fsn371004-bib-0024] Guvendiren, M. , H. D. Lu , and J. A. Burdick . 2012. “Shear‐Thinning Hydrogels for Biomedical Applications.” Soft Matter 8, no. 2: 260–272.

[fsn371004-bib-0025] Heikal, Y. A. R. , A. A. Hassan , A. A. Abou‐Arab , F. M. Abu‐Salem , and D. E.‐S. H. Azab . 2023. “Nano Formulated Soy Proteins as a Fat Replacer in Low Fat Mayonnaise Formula.” Journal of the Saudi Society of Agricultural Sciences 22, no. 7: 469–479.

[fsn371004-bib-0026] Hyrslova, I. , G. Krausova , I. Mrvikova , et al. 2022. “Functional Properties of Dunaliella Salina and Its Positive Effect on Probiotics.” Marine Drugs 20, no. 12: 781. 36547928 10.3390/md20120781 PMC9781844

[fsn371004-bib-0027] ISO‐3271 . 1975. ISO 3271:1975—Determination of Emulsion Stability of Protein. International Organization for Standardization (ISO).

[fsn371004-bib-0028] ISO‐6496 . 1983. ISO 6496:1983—Animal Feeding Stuffs—Determination of Moisture and Other Volatile Matter. International Organization for Standardization (ISO).

[fsn371004-bib-0029] ISO‐936 . 1998. ISO 936:1998—Animal and Vegetable Fats and Oils—Determination of Ash. International Organization for Standardization (ISO).

[fsn371004-bib-0030] Khalid, S. , K. Chaudhary , H. Aziz , et al. 2024. “Trends in Extracting Protein From Microalgae Spirulina Platensis, Using Innovative Extraction Techniques: Mechanisms, Potentials, and Limitations.” Critical Reviews in Food Science and Nutrition 65: 1–17. 10.1080/10408398.2024.2386448 39096052

[fsn371004-bib-0031] Kuhavichanan, A. , P. Kusolkumbot , S. Sirisattha , and C. Areeprasert . 2018. “Mechanical Extraction of Protein Solution From Microalgae by Ultrasonication.” IOP Conference Series: Earth and Environmental Science 109: 2009.

[fsn371004-bib-0032] Kumar, Y. , S. Roy , A. Devra , A. Dhiman , and P. K. Prabhakar . 2021. “Ultrasonication of Mayonnaise Formulated With Xanthan and Guar Gums: Rheological Modeling, Effects on Optical Properties and Emulsion Stability.” LWT 149: 111632.

[fsn371004-bib-0033] Martinez‐Cuazitl, A. , G. J. Vazquez‐Zapien , M. Sanchez‐Brito , et al. 2021. “ATR‐FTIR Spectrum Analysis of Saliva Samples From COVID‐19 Positive Patients.” Scientific Reports 11, no. 1: 19980. 34620977 10.1038/s41598-021-99529-w PMC8497525

[fsn371004-bib-0034] Metri‐Ojeda, J. , M. Ramírez‐Rodrigues , L. Rosas‐Ordoñez , and D. Baigts‐Allende . 2022. “Development and Characterization of a Low‐Fat Mayonnaise Salad Dressing Based on Arthrospira Platensis Protein Concentrate and Sodium Alginate.” Applied Sciences 12, no. 15: 7456.

[fsn371004-bib-0035] Mirzanajafi‐Zanjani, M. , M. Yousefi , and A. Ehsani . 2019. “Challenges and Approaches for Production of a Healthy and Functional Mayonnaise Sauce.” Food Science & Nutrition 7, no. 8: 2471–2484. 31428335 10.1002/fsn3.1132 PMC6694423

[fsn371004-bib-0036] Mohammadi, A. , S. A. Shahidi , A. Rafe , S. Naghizadeh Raeisi , and A. Ghorbani‐HasanSaraei . 2022. “Rheological Properties of Dairy Desserts: Effect of Rice Bran Protein and Fat Content.” Journal of Food Science 87, no. 11: 4977–4990. 36169930 10.1111/1750-3841.16339

[fsn371004-bib-0037] Mohammadi, S. , M. Alimi , S. A. Shahidi , and S. Shokoohi . 2024. “Investigating the Physicochemical, Rheological, and Sensory Properties of Low‐Fat Mayonnaise Prepared With Amaranth Protein as an Egg Yolk Replacer.” Food Science & Nutrition 12, no. 7: 5147–5161. 39055190 10.1002/fsn3.4163 PMC11266923

[fsn371004-bib-0038] Moreira, C. , P. Ferreira‐Santos , R. Nunes , et al. 2025. “Influence of Different Processing Techniques on Microalgal Protein Extraction.” Algal Research 86: 103958.

[fsn371004-bib-0039] Mosibo, O. K. , G. Ferrentino , and C. C. Udenigwe . 2024. “Microalgae Proteins as Sustainable Ingredients in Novel Foods: Recent Developments and Challenges.” Food 13, no. 5: 733. 10.3390/foods13050733 PMC10930894 38472846

[fsn371004-bib-0040] Nunes, E. , K. Odenthal , N. Nunes , T. Fernandes , I. A. Fernandes , and M. A. P. de Carvalho . 2024. “Protein Extracts From Microalgae and Cyanobacteria Biomass. Techno‐Functional Properties and Bioactivity: A Review.” Algal Research 82: 103638.

[fsn371004-bib-0041] Ouraji, M. , M. Alimi , A. Motamedzadegan , and S. Shokoohi . 2020. “Faba Bean Protein in Reduced Fat/Cholesterol Mayonnaise: Extraction and Physico‐Chemical Modification Process.” Journal of Food Science and Technology 57: 1774–1785. 32327788 10.1007/s13197-019-04211-9 PMC7171015

[fsn371004-bib-0042] Pagels, F. , and A. C. Guedes . 2023. “β‐Carotene From Dunaliella: Production, Applications in Food/Feed, and Recent Advances.” Handbook of Food and Feed From Microalgae 5: 203–219.

[fsn371004-bib-0043] Pandita, G. , S. Sharma , I. E. Oommen , et al. 2024. “A Comprehensive Review on the Potential of Ultrasound for Blue Food Protein Extraction, Modification and Impact on Bioactive Properties.” Ultrasonics Sonochemistry 111: 107087. 39362033 10.1016/j.ultsonch.2024.107087 PMC11480250

[fsn371004-bib-0044] Pispas, K. , G. Manthos , E. Sventzouri , et al. 2024. “Optimizing Phycocyanin Extraction From Cyanobacterial Biomass: A Comparative Study of Freeze–Thaw Cycling With Various Solvents.” Marine Drugs 22, no. 6: 246. 38921557 10.3390/md22060246 PMC11204620

[fsn371004-bib-0045] Rakipov, I. T. , A. A. Petrov , A. A. Akhmadiyarov , A. A. Khachatrian , and M. A. Varfolomeev . 2022. “FTIR Spectral Study of Intermolecular Interactions of C=O Groups of Amides in Solution.” Journal of Molecular Liquids 354: 118838.

[fsn371004-bib-0046] Savadkoohi, S. , and S. Kasapis . 2016. “High Pressure Effects on the Structural Functionality of Condensed Globular‐Protein Matrices.” International Journal of Biological Macromolecules 88: 433–442. 27060534 10.1016/j.ijbiomac.2016.04.012

[fsn371004-bib-0047] Schädle, C. N. , S. Bader‐Mittermaier , and S. Sanahuja . 2022. “Characterization of Reduced‐Fat Mayonnaise and Comparison of Sensory Perception, Rheological, Tribological, and Textural Analyses.” Food 11, no. 6: 806. 10.3390/foods11060806 PMC8954533 35327229

[fsn371004-bib-0048] Soltan, O. I. , H. S. Gazwi , A. E. Ragab , et al. 2023. “Assessment of Bioactive Phytochemicals and Utilization of *Rosa canina* Fruit Extract as a Novel Natural Antioxidant for Mayonnaise.” Molecules 28, no. 8: 3350. 37110582 10.3390/molecules28083350 PMC10146642

[fsn371004-bib-0049] Sousa, I. , L. Gouveia , A. P. Batista , A. Raymundo , and N. M. Bandarra . 2008. “Microalgae in Novel Food Products.” Food Chemistry Research Developments 2: 75–112.

[fsn371004-bib-0050] Srikanth, D. , D. Gopi , C. Sunil , K. Michael , and A. Rawson . 2023. “Proteins as Fat Replacers in the Food Industry.” Fat Mimetics for Food Applications 1: 133–154.

[fsn371004-bib-0051] Taslikh, M. , N. Mollakhalili‐Meybodi , A. M. Alizadeh , M.‐M. Mousavi , K. Nayebzadeh , and A. M. Mortazavian . 2022. “Mayonnaise Main Ingredients Influence on Its Structure as an Emulsion.” Journal of Food Science and Technology 59, no. 6: 2108–2116. 35602460 10.1007/s13197-021-05133-1 PMC9114219

[fsn371004-bib-0052] Uribe‐Wandurraga, Z. N. , I. Martínez‐Sánchez , C. Savall , P. García‐Segovia , and J. Martínez‐Monzó . 2021. “Microalgae Fortification of Low‐Fat Oil‐In‐Water Food Emulsions: An Evaluation of the Physicochemical and Rheological Properties.” Journal of Food Science and Technology 58: 3701–3711. 34471294 10.1007/s13197-020-04828-1 PMC8357875

[fsn371004-bib-0053] Wang, F. , Y. Li , C. R. Gough , Q. Liu , and X. Hu . 2021. “Dual‐Crystallizable Silk Fibroin/Poly (L‐Lactic Acid) Biocomposite Films: Effect of Polymer Phases on Protein Structures in Protein‐Polymer Blends.” International Journal of Molecular Sciences 22, no. 4: 1871. 33668676 10.3390/ijms22041871 PMC7918901

[fsn371004-bib-0054] Weidner, T. , N. F. Breen , G. P. Drobny , and D. G. Castner . 2009. “Amide or Amine: Determining the Origin of the 3300 Cm− 1 NH Mode in Protein SFG Spectra Using 15N Isotope Labels.” Journal of Physical Chemistry B 113, no. 47: 15423–15426. 19873996 10.1021/jp908773c PMC2783900

[fsn371004-bib-0055] Xia, E. , L. Zhai , Z. Huang , et al. 2019. “Optimization and Identification of Antioxidant Peptide From Underutilized *Dunaliella salina* Protein: Extraction, In Vitro Gastrointestinal Digestion, and Fractionation.” BioMed Research International 2019, no. 1: 6424651. 31531361 10.1155/2019/6424651 PMC6720044

[fsn371004-bib-0056] Yalmanci, D. , E. Dertli , Z. H. Tekin‐Cakmak , and S. Karasu . 2023. “The Stabilisation of Low‐Fat Mayonnaise by Whey Protein Isolate‐Microbial Exopolysaccharides (*Weissella confusa* W‐16 Strain) Complex.” International Journal of Food Science & Technology 58, no. 3: 1307–1316.

[fsn371004-bib-0057] Zakeri, S. M. , S. Shokoohi , and S. A. Shahidi . 2025. “Acetylated Distarch Adipate Quinoa Starch in Tomato Ketchup: Modification Process and Functional Charactristics.” Applied Food Research 1, no. 5: 100708.

[fsn371004-bib-0058] Zhang, W. , I. D. Boateng , and J. Xu . 2024. “How Does Ultrasound‐Assisted Ionic Liquid Treatment Affect Protein? A Comprehensive Review of Their Potential Mechanisms, Safety Evaluation, and Physicochemical and Functional Properties.” Comprehensive Reviews in Food Science and Food Safety 23, no. 1: e13261. 38284575 10.1111/1541-4337.13261

